# A prospective key informant surveillance system to measure maternal mortality – findings from indigenous populations in Jharkhand and Orissa, India

**DOI:** 10.1186/1471-2393-8-6

**Published:** 2008-02-28

**Authors:** Sarah Barnett, Nirmala Nair, Prasanta Tripathy, Jo Borghi, Suchitra Rath, Anthony Costello

**Affiliations:** 1 UCL Centre for International Health and Development, Institute of Child Health, University College London, 30 Guilford Street, London, WC1N 1EH, UK; 2 Ekjut, 107, Laxman Singh Complex-I, Munirka, New Delhi, Pin-11007, India; 3 Infectious Diseases Epidemiology Unit, Department of Epidemiology and Population Health, London School of Hygiene & Tropical Medicine, Keppel Street, London, WC1E 7HT, UK

## Abstract

**Background:**

In places with poor vital registration, measurement of maternal mortality and monitoring the impact of interventions on maternal mortality is difficult and seldom undertaken. Mortality ratios are often estimated and policy decisions made without robust evidence. This paper presents a prospective key informant system to measure maternal mortality and the initial findings from the system.

**Methods:**

In a population of 228 186, key informants identified all births and deaths to women of reproductive age, prospectively, over a period of 110 weeks. After birth verification, interviewers visited households six to eight weeks after delivery to collect information on the ante-partum, intra-partum and post-partum periods, as well as birth outcomes. For all deaths to women of reproductive age they ascertained whether they could be classified as maternal, pregnancy related or late maternal and if so, verbal autopsies were conducted.

**Results:**

13 602 births were identified, with a crude birth rate of 28.2 per 1000 population (C.I. 27.7–28.6) and a maternal mortality ratio of 722 per 100 000 live births (C.I. 591–882) recorded. Maternal deaths comprised 29% of all deaths to women aged 15–49. Approximately a quarter of maternal deaths occurred ante-partum, a half intra-partum and a quarter post-partum. Haemorrhage was the commonest cause of all maternal deaths (25%), but causation varied between the ante-partum, intra-partum and post-partum periods. The cost of operating the surveillance system was US$386 a month, or US$0.02 per capita per year.

**Conclusion:**

This low cost key informant surveillance system produced high, but plausible birth and death rates in this remote population in India. This method could be used to monitor trends in maternal mortality and to test the impact of interventions in large populations with poor vital registration and thus assist policy makers in making evidence-based decisions.

## Background

Maternal mortality in the developing world remains high, with little progress towards Millennium Development Goal (MDG) 5 [1,2]. There remains little evidence of the impact of interventions on maternal mortality at the population level in poor and remote areas, where ratios are often highest. The main challenge in effectively monitoring progress and the impact of interventions is the difficulty in measuring maternal mortality.

Maternal death is a relatively rare event, so large sample sizes are needed to monitor mortality ratios. Conventional surveillance systems are expensive and logistically challenging and consequently, there have only been three published trials that have attempted to measure population maternal mortality ratio [3-5]. Furthermore, under-reporting is frequent with most deaths occurring outside of the health system and in countries without efficient vital registration systems. Cause of death assignment is difficult: where death certificates are completed they do not stipulate pregnancy status [6,7] and maternal deaths may be intentionally misclassified [8]. Thus there is a great need for alternative methods for measuring maternal mortality [9].

India has the largest number of maternal deaths in the world and accounts for 22% of all maternal deaths [10]. About two thirds of these occur in just nine states, which include Jharkhand and Orissa [11]. There are an estimated 90 million indigenous people in India, referred to as "scheduled tribes" or "adivasis". Research has shown that an indigenous person in India is 1.2 times more likely to experience excess mortality compared to a non-indigenous person with the same standard of living [12].

This paper presents a prospective key informant system of birth and death identification that is designed to be cheaper and simpler than conventional surveillance systems. It aims to: measure crude birth rates and maternal mortality in a remote, predominantly indigenous population in eastern India; identify pregnancy related and late maternal deaths; ascertain the breakdown of maternal deaths by cause and determine the timing and place of death; and report the cost of the system.

## Methods

A key informant surveillance system was established in three contiguous districts in two states in India; West Singhbhum and Saraikela-Kharswan in Jharkhand and Keonjhar in Orissa. Within each district, twelve population clusters were selected; the average population per cluster is 6338, covering a total population of 228,186. The clusters are largely physically remote from health services and comprise a high proportion (73%) of indigenous people. Other inhabitants are largely from the scheduled (3%) and other backward castes (24%).

**Figure 1:**
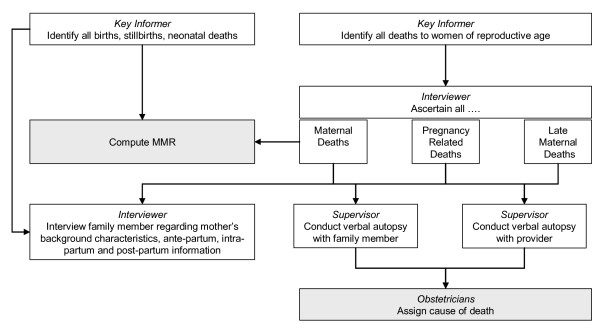
Maternal Mortality Key Informant Surveillance System.

The measurement of maternal mortality is just one component of a larger key informant surveillance system designed to identify all births, neonatal and maternal outcomes for the duration of a randomised controlled trial. The whole system is described below but this paper will just present results and costs for the maternal mortality component. The system comprises two stages; the *identification stage *and the *interviewing stage *(Figure 1).

The *identification stage *utilised local key informants toidentify all births and deaths to women of reproductive age (15–49). Most key informants were traditional birth attendants (TBAs). One key informant was recruited to cover a geographic area of approximately 250 households. They were paid an incentive of 30 rupees ($0.65) for every accurate birth or death identification. The key informants met with an interviewer two-three times a month to pass on the relevant information and the interviewer in turn visited the relevant households to verify the births and deaths before paying the identifiers. The key informers only missed one late maternal death where a woman had migrated to work at a brick kiln. In this instance the local auxillary nurse midwife informed the interviewer.

The *interviewing stage *utilised one full-time salaried interviewer for each cluster. Their role was to verify the information provided by key informants; to interview all mothers six weeks after their delivery to collect detailed information on the ante-partum, intra-partum, and post-partum periods, as well as background information; and to interview family members in the case of a death of a women of reproductive age to ascertain whether she was pregnant, or had recently given birth. The interviewer ascertained whether a death was maternal, late maternal, pregnancy related or none of these by interviewing relatives of the deceased, without the use of a verbal autopsy tool. Eighty two per cent of all interviews were conducted between six to eight weeks after delivery.

One monitoring supervisor per district supervised the surveillance system. In the case of a maternal, pregnancy related or late maternal death they conducted verbal autopsies with a close friend or relative of the mother, ideally present at the time of death. 47 verbal autopsies were with husbands; 55 with other relatives and one with a friend. 94 of the 103 respondents were present at the time of death. Supervisors also tried to conduct verbal autopsies with formal and informal care providers, present at the time of death, but these proved more difficult and only seventeen out of a possible 37 providers were interviewed. 70% of maternal verbal autopsies were conducted within eight weeks after delivery.

Verbal autopsies were reviewed independently by two local obstetricians from the study area. There was agreement on primary causation in all but seven cases. In the case of disagreement a third doctor was consulted.

Finally, the costs of the surveillance system incurred by the project were explored. Finanacial data were obtained from project accounts. Costs were classified in terms of: identification of births and deaths; eliminating deaths to women of reproductive age that were not maternal, late maternal or pregnancy related by interviewers; interviews, verbal autopsies; and data management (including database design, data entry by data entry clerk and data analysis by a number of project staff, and associated equipment). The cost of verbal autopsies included the cost of the supervisor and obstetricians time assigning cause of death. Although, the obstetricians volunteered their time, and were not paid by the project, their time was valued using average salary rates as, in the case of scale-up to a larger population, an incentive payment may be necessary. Costs are presented as average monthly costs and as unit costs (e.g. cost per identification). The costs of setting up the system in terms of staff recruitment and training were also estimated. Costs are presented in 2006 US dollars ($1= IRS 44.1).

The surveillance system is on-going as it is being used to monitor the impact of a randomized controlled trial, and this paper refers to births occurring in the first 110 weeks, from 21^st ^November 2004 to 31^st ^Dec 2006. Maternal and pregnancy related deaths may occur up to 42 days after the birth and are therefore included up until 11^th ^February. Late maternal deaths may occur up to one year after the birth and have been followed up until 31^st ^December 2007.

## Results

### Birth rates and maternal outcomes

In a population of 228 186, over a 110 week period, 13 602 births were identified, of which 13 160 were live births (Table 1). The crude birth rate is 28 per 1000 population. 323 deaths to women of reproductive age were identified, of these 29% (95) were classified as maternal deaths, giving a maternal mortality ratio (MMR) of 722 per 100 000 live births (CI 591–882). Four additional pregnancy related and four late maternal deaths were identified.

**Table 1:** Birth rates and maternal outcomes

		95% Confidence Interval
Total Population	228,186	
Number of births	13602	
Crude birth rate per 1000 population	28.2	(27.7–28.6)
Number of live births	13160	
		
Number of deaths to women of reproductive age (15–49)	323	
Number of maternal deaths	95	
Number of pregnancy related deaths	99	
Number of late maternal deaths*	4	
		
Maternal mortality ratio per 100 000 live births (n)	722	(591–882)

### Cause and timing of maternal deaths

A quarter of all maternal deaths (24%) occurred during the ante-partum period, half during the intra-partum period and up to 48 hours afterwards (48%) and just over a quarter up to 42 days post-partum (27%) (Table 2).

**Table 2:** Primary causes of maternal deaths by time of death

Primary cause of death	Ante-partum		Intra-partum (< 48 hours)		Post-partum (< 42 days)		Total	
	
	%	(n)	%	(n)	%	(n)	%	(n)
All Deaths	24.2	(23)	48.4	(46)	27.4	(26)		
								
Haemorrhage	8.7	(2)	39.1	(18)	15.4	(4)	25.3	(24)
Malaria	47.8	(11)	10.9	(5)	23.1	(6)	23.2	(22)
Sepsis	4.3	(1)	13.0	(6)	34.6	(9)	16.8	(16)
Hypertensive disorder	21.7	(5)	10.9	(5)	11.5	(3)	13.7	(13)
Obstructed Labour			17.4	(8)			8.4	(8)
Anaemia					3.8	(1)	1.1	(1)
Embolism					7.7	(2)	2.1	(2)
Abortion	8.7	(2)					2.1	(2)
Other direct*			6.5	(3)	3.8	(1)	4.2	(4)
Other indirect**	8.7	(2)	2.2	(1)			3.2	(3)

n = 95								

* Retained placenta and obstetric shock

** Tuberculosis and heart disease

Haemorrhage was the primary cause of death for a quarter of maternal deaths (25%); closely followed by malaria (23%); and sepsis (17%). Malaria was the most common cause of death during the ante-partum period (48%); haemorrhage during the intra-partum period (39%); and sepsis during the post-partum period (35%). Secondary causes were also identified from the verbal autopsies. Anaemia was a key underlying factor in 35% of deaths. Malaria (22) and Sepsis (20) were also highlighted as key underlying causes.

The additional pregnancy related deaths were due to suicide, homicide, thyroid cancer and lightening and the late maternal deaths were due to malaria, sepsis, embolism, and tuberculosis.

220 additional deaths to women of reproductive age were identified. The most common causes of death were malaria (46), tuberculosis (28), accidental causes (29), anaemia (22), gastro-intestinal conditions (20) and suicide (15).

#### Place of delivery and death

85% of all of the deliveries took place at home, 15% took place in a facility and 0.1% in transit. For those who died the percentages were very similar with 87% of the deliveries occurring at home and 13% in a facility.

60% of deaths occurred at home, 28% in a facility, 6% in transit to a facility and 5% in transit from a facility (Table 3). There was very little variation in the place of death, when broken down by timing with a similar pattern seen during the ante-partum, intra-partum, and post-partum periods.

**Table 3:** Place of death by time of death and place of delivery

	Place of death									
	
	Home		Facility		Transit to facility		Transit from facility		Field	
	
	%	(n)	%	(n)	%	(n)	%	(n)	%	(n)
All Deaths	60.2	62	28.2	29	5.8	6	4.9	5	1	1
										
Time of Death										
Ante-partum	60.0	15	32.0	8	8.0	2				
Intra-partum (< 48 hours)	58.7	27	21.7	10	8.7	4	10.9	5		
Postpartum (< 42 days)	62.5	15	37.5	9						
Late maternal deaths (42 days – 1 year)	50.0	2	50.0	2						
Pregnancy related	75.0	3	0						25.0	1
										
Place of delivery										
Home	68.3	41	23.3	14	3.3	2	3.3	2	1.7	1
Facility			88.9	8			11.1	1		
Died during pregnancy	61.8	21	20.6	7	11.8	4	5.9	2		

n = 103										

For those who delivered at home and died, 68% died at home and 23% made it to a facility, while 3% died in transit to a facility and 3% in transit from a facility. For those who delivered in a facility and died, most died in a facility (89%) while 11% died in transit from the facility. For those who died during pregnancy 62% died at home, 21% at a facility, 12% in transit to a facility.

#### Cost of surveillance system

The average monthly cost of identification was US$ 386 (US$ 0.70 per identification) (Table 4). The average monthly cost of the monitors eliminating deaths to women of reproductive age that were not maternal, late maternal or pregnancy related deaths was US$ 9 (US$ 1 per recording). The average monthly cost of interviews was US$ 2,311 (US$ 0.17 per interview) and the average cost of verbal autopsies was US$ 54 (US$ 0.44 per VA). The average monthly cost of data management and analysis was US$ 343. The average monthly cost overall was US$ 2,759 (US$ 0.20 per birth interviewed), or US$ 3,103 inclusive of data management (US$ 0.23 per birth interviewed). Staff represented 84% of the cost; transport 9% and supplies 5%. The one-off cost of recruiting and training all staff was US$ 7,355.

**Table 4:** Breakdown of the costs of the surveillance system by activity and resource

Cost component	Identification	Eliminating non-maternal deaths	Interviews	Verbal Autopsies	Total
Key informants	375	-	-	-	375
Interviewers	-	6.83	1 690	-	1 697
Supervisers	-	0.88	219	25	245
Obstertician	-	-	-	11	11
Supplies *(Printing, stationary)*	-	0.55	135	-	136
Supplies *(Hats, badges, bags)*	11	-	-	-	11
Communication	-	0.09	23	1	24
Refreshments	-	0.03	7	0	7
Transport	-	0.96	238	16	255

Total	386	9.34	2 311	54	2 759

## Discussion

This study has shown that even in remote, deprived populations a key informant system can produce reliable and plausible maternal mortality ratios at low cost. The key informant method has several important advantages. The system is designed to prospectively measure, rather than estimate, levels of maternal mortality in a given population. Most maternal mortality data for developing countries are estimates, derived retrospectively, from censuses, facility records and indirect methods such as adding questions to household or Demographic and Health Surveys (DHS). The 'sisterhood method' generates retrospective data and attempts to overcome sample size problems by asking all adult women in a household questions about the survival of their sisters, but often excludes information on the cause or circumstances surrounding the death [13] 'Networking' tries to further address sample size problems by asking women if they are aware of any women who died from maternal causes in the preceding year [14]. To avoid missing maternal deaths the key informant system identifies all deaths to women of reproductive age and subsequently exposes maternal deaths through a process of elimination. In this respect this system could be considered to be a prospective variant of 'Reproductive Age Mortality Surveys'. This has the further benefit that it can also provide useful information about non-maternal deaths to women of reproductive age if desired. Furthermore, since the key identifiers identify all births and neonatal outcomes this process also provides an accurate denominator.

Few Safer Motherhood programmes or research trials attempt to measure maternal mortality directly due to the large sample size required and the perceived high cost involved. The key informant system is acceptable, feasible and affordable for epidemiological surveillance of research or development projects. Mothers were only visited once, six to eight weeks after delivery. Compared with methods requiring frequent visits [4], which could potentially result in higher refusal rates, our system reduces costs and is less intrusive to families. The system may be seen as similar to conventional demographic surveillance systems, but differences include the use of incentivised key informants (who are not health workers and do not routinely visit every household in a defined area); the application across a large number of dispersed clusters; and the relatively low cost. The use of an incentive driven system avoids ethical dilemmas associated with the use of unpaid volunteers, while remaining low cost. The essential cost, in terms of deriving an estimate of the maternal mortality ratio is simply the cost of identification of deaths and verification, a monthly cost of US$386 in our area, or US $0.02 per capita per year.

Concerns that key informers may over report births or deaths to earn more money are invalid, as payment is only made once an identification has been verified by interviewers. In regards to missing births or deaths, the informants were allocated manageable geographical areas, based on whole hamlets or distinct parts of a village, which are familiar and accessible, minimising the risk of any births or deaths being missed. The death of any woman of reproductive age is a significant event that an informant living in the community would naturally be aware of, enabling deaths during pregnancy and late maternal deaths to be captured. However, migration does pose a problem.

The results presented in this paper are for women of the standard reproductive age group 15–49 years. In the study all deaths to women aged 10–50 are recorded to ensure there is no under-reporting of maternal deaths due to age restrictions, as early marriage is common in this setting and many people do not know their exact age. However, during the 110 week study period no maternal deaths have been found for this age-group and hence results presented in this paper are just for those aged 15–49.

This method for measuring maternal mortality appears to be robust, but as with all methods for measuring maternal mortality in the community there are clearly some limitations. Maternal deaths could be intentionally misclassified, especially with regards to unsafe abortion. There are a lot of suicides reported in this population, and the relatives of two women who died due to menstrual problems refused to give a detailed interview. It is possible that some of these women may have been pregnant or had unsafe abortions. Furthermore, using verbal autopsies to assign the primary cause of death is difficult when no formal provider is present. There was some uncertainty over assigning malaria, sepsis or anaemia as a cause of death without the availability of test results. Delays in collecting data could also lead to recall problems. The sample size of 13660 births, though large, may not be considered enough for precise maternal mortality measurement. However, an analysis of the MMR at 60 weeks from 7500 births produced a very similar MMR. Over time, or with a larger population, the increase in sample size would add to the robustness of the mortality measurements.

The findings from this surveillance system raise important issues for policymakers and health professionals. The system has produced high, but plausible birth and death rates in this rural, predominantly indigenous, population in India. Our observed crude birth rate of 28 births per 1000 population are consistent with state level reports of 29 in Jharkhand and 24 in Orissa [15]. The MMR of 722 per 100 000 live births was alarmingly high for a population unaffected by HIV. The most recent estimate of the MMR for India is 450 per 100 000 live births [10]. The MMR for Orissa has been reported as 367 per 100,000 live births [16]. No official figures are currently available for MMR for Jharkhand (independent of Bihar), but the state government has indicated that its goal for the MMR for 2006–07 was 407 per 100,000 live births and 325 for 2009–10 [17]. The high levels of mortality in our study area probably relate to socioeconomic deprivation in indigenous populations in remote areas in India and highlight a pressing need for interventions targeted at improving maternal and newborn care in these vulnerable populations.

The tenth version of the International Classification of Disease recommended two new additions with regard to measuring maternal mortality; pregnancy related deaths and late maternal deaths [18]. It is debatable whether the suicide and homicide deaths should be included as maternal deaths. There is mounting evidence that being pregnant may place women at greater risk of dying from suicide and homicide, and that unwanted pregnancies might be an important factor for the increased risk [19]. The inclusion of these two deaths from homicide and suicide would raise the MMR from 722 to 737 and including all pregnancy related deaths would raise it to 752 (i.e. remaining within the current confidence interval). This small difference suggests that pregnancy related deaths could be a useful proxy indicator for maternal deaths in similar contexts where cause of death data is unavailable. This supports the argument that it would be worthwhile including information on death certificates stipulating pregnancy status [20] to help measure maternal mortality. This system also enables the identification of late maternal deaths. One justification for this category is that modern life-sustaining procedures can prolong dying and delay death. This data show that although late maternal deaths even occur in a context with very limited access to quality health care, most maternal deaths did take place within the time boundaries of the current definition.

It has been estimated that two-thirds of maternal deaths occur in late pregnancy through to 48 hours after delivery [6]. This data showed a higher proportion (52%) of deaths occurring outside the intra-partum period, suggesting that there may be considerable variability in the contribution of the intra-partum period to maternal mortality in different contexts. Therefore policies solely focusing on interventions targeting this period may be less appropriate for vulnerable and excluded populations. Admittedly, some of those problems occurring in the postpartum period may be prevented by interventions in the intra-partum period, especially sepsis, and the higher proportion of deaths occurring outside the intra-partum period may be partly attributable to the high prevalence of malaria within this context.

The data show that in this remote rural population, most maternal deaths took place at home (60%) rather than in a facility (28%). Other studies suggest that most maternal deaths occur in facilities, but this evidence relies heavily on facility records or studies from countries with a higher proportion of facility deliveries rather than population data from poor rural communities [20]. It is also feasible that deaths at home in similar contexts are more likely to be missed by conventional methods for monitoring maternal mortality.

## Conclusion

The difficulties in measuring maternal mortality are a frequent constraint to having accurate measurements and monitoring the impact of interventions. Hence there is a need for alternative approaches and this paper proposes a key informant surveillance system. The system has provided valuable and reliable prospective data on maternal mortality in a remote indigenous population in India. This approach to measuring maternal mortality should allow governments and researchers to take an evidence-based approach to evaluate programmes and strategies for maternal mortality reduction in even the most vulnerable and remote communities in the developing world. Similar sentinel sites in developing countries could produce reliable MMRs and monitor the impact of large scale interventions. If the method works well in this poor, remote indigenous area with very limited infrastructure and low levels of education, then it could be replicated almost anywhere.

## Competing interests

The author(s) declare that they have no competing interests.

## Authors' contributions

All the authors contributed to the design of the study and criticised drafts of the paper. PT, NN, SB and AC were responsible for the conception and overall supervision of the project. PT and NN managed the project, data collection, data entry and administration. SB and AC were technical advisers to the study. JB provided technical assistance with the collection and analysis of cost data. SB designed the key informant surveillance system, the original trial protocol, data collection tools and wrote the first draft of the paper. SB and NN carried out the quantitative analysis. SB, NN, PT, JB, SR and AC were responsible for subsequent collation of inputs and redrafting. PT and AC will stand as guarantors for the paper.

## Pre-publication history

The pre-publication history for this paper can be accessed here:


